# PPARα agonist fenofibrate prevents postoperative cognitive dysfunction by enhancing fatty acid oxidation in mice

**DOI:** 10.1515/tnsci-2022-0317

**Published:** 2023-11-15

**Authors:** Tiantian Liu, Xinlu Chen, Ziqi Wei, Xue Han, Yujia Liu, Zhengliang Ma, Tianjiao Xia, Xiaoping Gu

**Affiliations:** Department of Anesthesiology, Nanjing Drum Tower Hospital, Affiliated Hospital of Medical School, Nanjing University, No. 321 Zhongshan Road, Nanjing 210008, China; Medical School, Nanjing University, Nanjing 210093, China; Jiangsu Key Laboratory of Molecular Medicine, Nanjing University, Nanjing, China; State Key Laboratory of Pharmaceutical Biotechnology, Nanjing University, Nanjing, China

**Keywords:** postoperative cognitive dysfunction, isoflurane, PPARα, fenofibrate, fatty acid oxidation

## Abstract

**Background:**

Due to high rates of incidence and disability, postoperative cognitive dysfunction (POCD) currently receives a lot of clinical attention. Disturbance of fatty acid oxidation is a potential pathophysiological manifestation underlying POCD. Peroxisome proliferator-activated receptor α (PPARα) is a significant transcription factor of fatty acid oxidation that facilitates the transfer of fatty acids into the mitochondria for oxidation. The potential role of PPARα intervention in POCD warrants consideration.

**Objective:**

The present study is aimed to investigate whether PPARα agonist fenofibrate (FF) could protect long-term isoflurane anesthesia-induced POCD model and to explore the potential underlying function of fatty acid oxidation in the process.

**Methods:**

We established the POCD model via 6 h long-term isoflurane anesthesia *in vivo* with C57BL/6J mice and *in vitro* with N2a cells. Cells and mice were pretreated with PPARα agonist FF before anesthesia, after which fatty acid oxidation and cognitive function were assessed. The level of fatty acid oxidation-related proteins was determined using western blotting. The contextual fear conditioning test was utilized to evaluate mice’s learning and memory.

**Results:**

Our results showed that 6 h long-term isoflurane anesthesia induced contextual memory damage in mice, accompanied by decreases of fatty acid oxidation-related proteins (peroxisome proliferator-activated receptor γ coactivator 1α, carnitine palmitoyltransferase 1A, and PPARα) both in the hippocampus of POCD mice and in N2a cells. In the N2a cell model, pretreatment of PPARα agonist FF led to the upregulation of fatty acid oxidation-related proteins. *In vivo* results showed that preconditioned FF reached similar effects. More crucially, FF has been shown to reduce cognitive damage in mice after long-term isoflurane anesthesia. Additionally, our data showed that after blocking fatty acid oxidation by Etomoxir, FF failed to protect cognitive function from long-term isoflurane anesthesia.

**Conclusions:**

Pretreatment of PPARα agonist FF can protect against long-term isoflurane anesthesia-induced POCD by enhancing fatty acid oxidation.

## Graphical abstract

**Figure:**
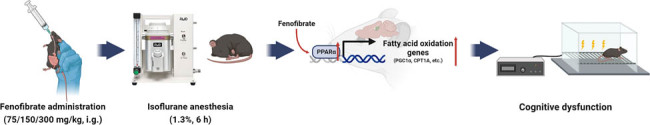

## 1 Introduction

Postoperative cognitive dysfunction (POCD) is a common neurological complication characterized by memory impairments and personality changes following anesthesia and surgery. Most studies concerning the mechanisms of POCD have focused on inflammatory response, metabolic disturbance, oxidative stress, etc. [1]. The metabolic syndrome is a typical metabolic disorder and its pathophysiology is largely attributable to insulin resistance [2]. Consistently, previous studies in our laboratory have shown that long-term isoflurane exposure induced peripheral and central insulin resistance in adult mice [3,4]. It is generally recognized that the development of these pathologies is associated with disorders of lipid metabolism [5]. Particularly, both animal experiments and clinical studies suggest that anesthesia and surgery can disrupt normal lipid metabolism processes [6,7]. Fatty acid oxidation is an important process for maintaining brain lipid metabolism homeostasis and represents up to 20% of brain oxidative energy production [8]. It is a complex process regulated by multiple genes, among which peroxisome proliferator-activated receptor (PPAR) is a crucial transcription factor that facilitates the transfer of fatty acids into the mitochondria for oxidation, containing three different isoforms (PPARα, PPARβ/δ, and PPARγ).

Roy et al. showed that PPARα-deficient but normal PPARβ or PPARγ hippocampal neurons had decreased calcium inflow and the expression of various synaptic plasticity-related molecules was low [9]. PPARα, the first member of the PPAR family to be cloned, is distributed in multiple brain regions in adults and rodents, including the thalamus, hypothalamus, hippocampus, basal ganglia, etc. [10]. PPARα can be activated by many ligands, including endogenous fatty acids and exogenous peroxisome proliferators [11]. Recent studies demonstrate that activation of PPARα has a variety of pharmacological effects on the central nervous system such as maintaining hippocampal neurogenesis and preventing memory impairment caused by global cerebral ischemia in rats, and the neuroprotective effect on Alzheimer’s disease (AD) and Parkinson’s disease (PD) [12,13].

Our study focused on whether the fatty acid oxidation process was involved in the isoflurane-induced POCD and showed the effect of increased PPARα levels on fatty acid oxidation and cognitive function. We administered PPARα agonist fenofibrate (FF) to mice as pharmacological intervention. As a lipid-lowering agent, FF is a fibric acid derivative that has been widely used for the treatment of dyslipidemia in the clinic [14,15]. The PPARα agonist FF pretreatment improved disorders of fatty acid oxidation and cognitive function in POCD mice.

## 2 Materials and methods

### 2.1 Animals

All animal experiments were performed with the approval of the Laboratory Animal Ethics Committee of Drum Tower Hospital, and all experimental procedures complied with the ARRIVE guidelines and were conducted according to the EU Directive 2010/63/EU for animal experiments. In this study, 8-week-old male C57BL/6J mice weighing 20–22 g were used. We tried our best efforts to minimize the number of mice. All animals adapted for at least 3 weeks, with a 12 h light/dark cycle (light on at 8:00 and light off at 20:00) and appropriate room temperature (23 ± 1°C). The animals were supplied with freely available food and water.

### 2.2 Drug treatment

Sodium carboxymethyl cellulose (CMC, 1%) (Selleckchem, USA) was prepared with saline. FF (Sigma, USA) was dissolved in 1% CMC to obtain 75, 150, and 300 mg/kg FF solutions. Mice randomized for FF pretreatment were injected 0.01 mL FF solution per gram intragastrically 30 min before anesthesia. The mice in the Con group and the Ane group were administered with the same volume of 1% CMC. Due to its solubilization property and stability, CMC solution is used to dissolve poorly soluble medicines or deliver medicines as a pharmaceutical carrier [16]. Also, our preliminary experiments have shown that 1% CMC did not impact mice’s cognitive disorders induced by isoflurane. ETO solution (25 mg/kg) was prepared using a 37°C water bath heating to dissolve FF with saline. We injected 0.01 mL/g intragastrically for 3 days before anesthesia.

### 2.3 General anesthesia

Mice were put in Plexiglass chambers prefilled with 5% isoflurane (Tocris, 9A/180370) in 100% oxygen (2–3 L/min) for induction. They were maintained under a mixture of 1.3% isoflurane and 100% oxygen. The anesthesia operation lasted 6 h. During the whole procedure, the inspiratory concentration of isoflurane and the respiratory activity of mice were monitored automatically or manually.

### 2.4 Fear conditioning test

Panlab Fear Conditioning System (Harvard Apparatus, Spain) was used to conduct the contextual fear conditioning test. During the training phase, mice were positioned in the chamber and were allowed to explore freely for 3 min, followed by a 30 s pulsing tone (80 dB, 2,000 Hz) and a 2 s mild foot shock (0.75 mA). Then, the mice remained in the chamber for 1.5 min. After 24 h, mice were positioned in the same chamber for 5 min without sound or shock for the contextual memory test. Throughout the test, Packwin 2.0 software was used to record the mice’s freezing behavior continually. The mice’s freezing behavior, an absolutely immovable posture except for breathing, was transformed to the percentage of freezing time to evaluate their learning and memory.

### 2.5 Cell culturing

Mouse neuroblastoma cells (N2a cells) were plated on 24 tissue culture well plates (Falcon, country) at 200,000 cells/well for 48 h in the tissue culture medium (Dulbecco’s modified Eagle’s medium [Thermo Fisher Scientific, Australia], 10% fetal bovine serum [Gibco, Fisher Scientific, Australia], and 1% penicillin/streptomycin [Sigma, USA]) at 37°C in 5% CO_2_ until optimum growth, and adhesion to the surface of the plates were observed. The medium was changed daily. After 48 h, cells were then trypsin-removed from the plates, centrifuged at 800 rpm for 5 min, and lysed with NP-40 lysis buffer (150 mM NaCl, 1.0% Nonidet P-40 and Triton X-100, 50 mM Tris-HCl; the pH was adjusted to 7.4) with the addition of an AEBSF protease inhibitor (Sigma, USA) and stored at −80°C until further use.

### 2.6 Western blot (WB)

To measure fatty acid oxidation-related protein levels (peroxisome proliferator-activated receptor γ coactivator 1α [PGC1α], carnitine palmitoyltransferase 1A [CPT1A], and PPARα), WB was performed. All mice were killed promptly following the behavioral test. The mice’s hippocampus was isolated and the tissue was stored at −80°C. In brief, the hippocampus tissue was washed and the surface water was dried. Then, it was homogenized in cold RIPA lysis buffer (Beyotime, China) with proteinase and phosphatase inhibitors (EMD Millipore, USA) and centrifuged to obtain protein supernatants. The total amount of protein was determined by the BCA protein method (Beyotime, China). The same amount of protein (40 μg) was loaded into each lane of 10% SDS-PAGE gel, separated by electrophoresis. Subsequently, the proteins were transferred to the polyvinylidene fluoride membrane (EMD Millipore, USA). After that, the membrane was blocked with 5% (w/v) milk for 1 h. The membranes were incubated with primary antibodies against PGC1α (Proteintech, USA), CPT1A (Proteintech, USA), PPARα (Proteintech, USA), β-actin (Abcam, USA), and GAPDH (Abcam, USA) for 1 h, followed by HRP-conjugated secondary antibodies (Elabscience, USA).

### 2.7 Statistical analyses

All data were expressed as mean ± SD (standard deviation). Statistical analyses of both cognitive behavioral tests and WBs were conducted by *t*-test or one-way ANOVA followed by Bonferroni post hoc tests using SPSS 17.0 software. Significance was defined when *P <* 0.05.


**Ethical approval:** The research related to animals’ use has been complied with all the relevant national regulations and institutional policies for the care and use of animals. All animal experiments were performed with the approval of the Laboratory Animal Ethics Committee of Drum Tower Hospital, and all experimental procedures complied with the ARRIVE guidelines and were conducted according to the EU Directive 2010/63/EU for animal experiments.

## 3 Results

### 3.1 Effects of long-term isoflurane anesthesia on contextual memory and fatty acid oxidation-related proteins in mice

**Figure 1:**
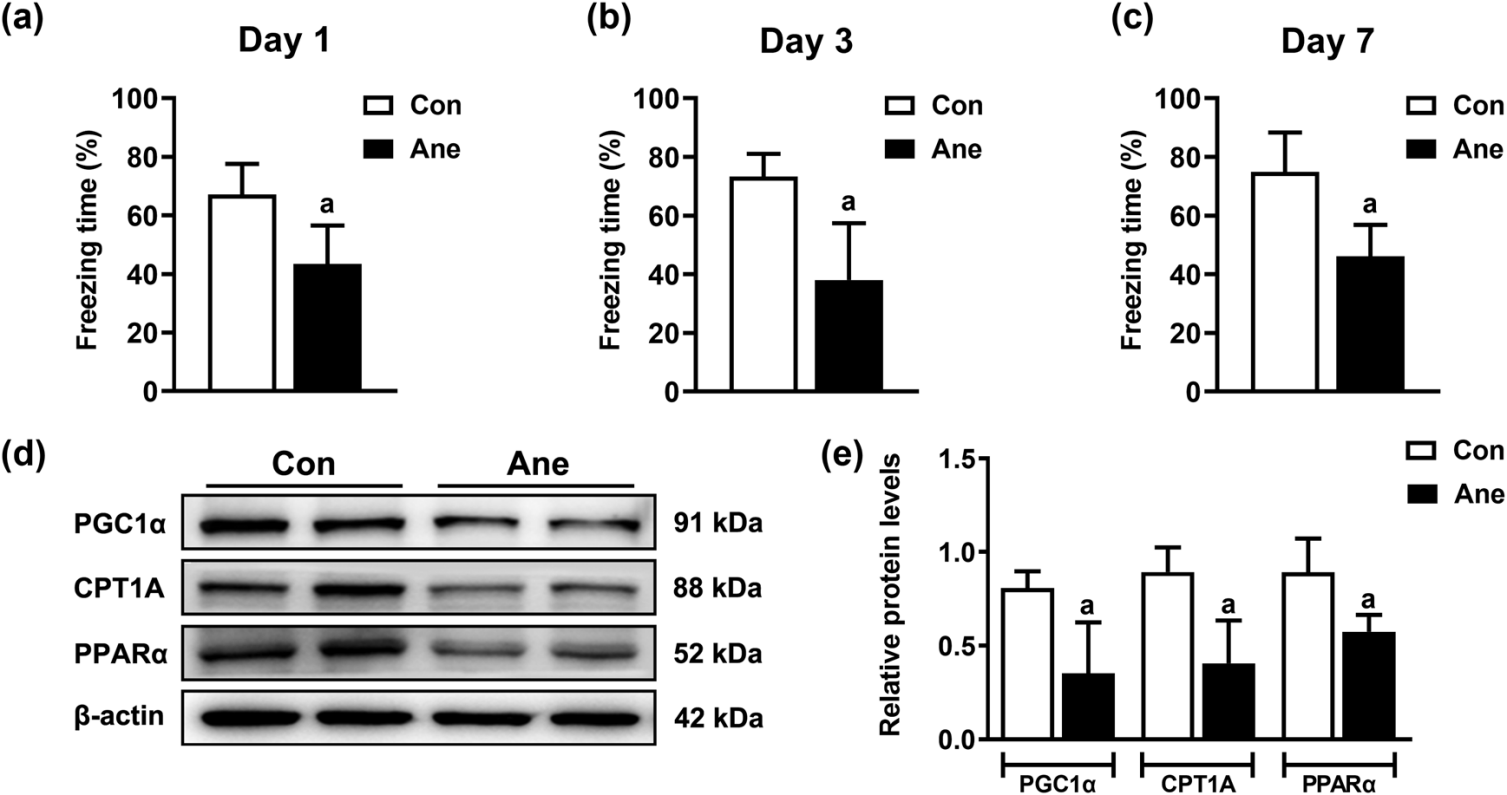
Long-term isoflurane anesthesia led to impaired cognition and reduced levels of fatty acid oxidation-related proteins in mice. Long-term isoflurane anesthesia leads to contextual memory impairments in mice on Day 1 (a), Day 3 (b), and Day 7 (c). All data are presented as mean ± SD (*n* = 6/group). ^a^ *P* < 0.05, compared with the Con group. (d) Long-term isoflurane anesthesia leads to a decreased level of fatty acid oxidation-related proteins PGC1α, CPT1A, and PPARα in mice. (e) The protein quantitation histogram of (d). All data are presented as mean ± SD (*n* = 4/group). ^a^ *P* < 0.05, compared with the Con group.

The animal model of POCD in C57BL/6J mice was established through 6 h isoflurane anesthesia-induced cognitive impairment. As shown in Figure 1a–c, we used a fear conditioning test to evaluate changes in cognitive function in POCD mice. In the contextual fear conditioning test, the percentage of freezing time of mice after anesthesia decreased remarkably on Day 1 (^a^ *P =* 0.006), Day 3 (^a^ *P =* 0.002), and Day 7 (^a^ *P =* 0.002) compared with the Con group. Meanwhile, to test the effects on the fatty acid oxidation pathway in POCD mice, the levels of fatty acid oxidation-related proteins PGC1α, CPT1A, and PPARα in the hippocampus of mice were measured using WB. Compared with the Con group, the protein levels of PGC1α (^a^ *P =* 0.019), CPT1A (^a^ *P =* 0.010), and PPARα (^a^ *P* = 0.020) in the hippocampus of the Ane group were significantly reduced after anesthesia (Figure 1d and e).

### 3.2 Effects of isoflurane and FF treatment on the fatty acid oxidation-related proteins in N2a cells

**Figure 2:**
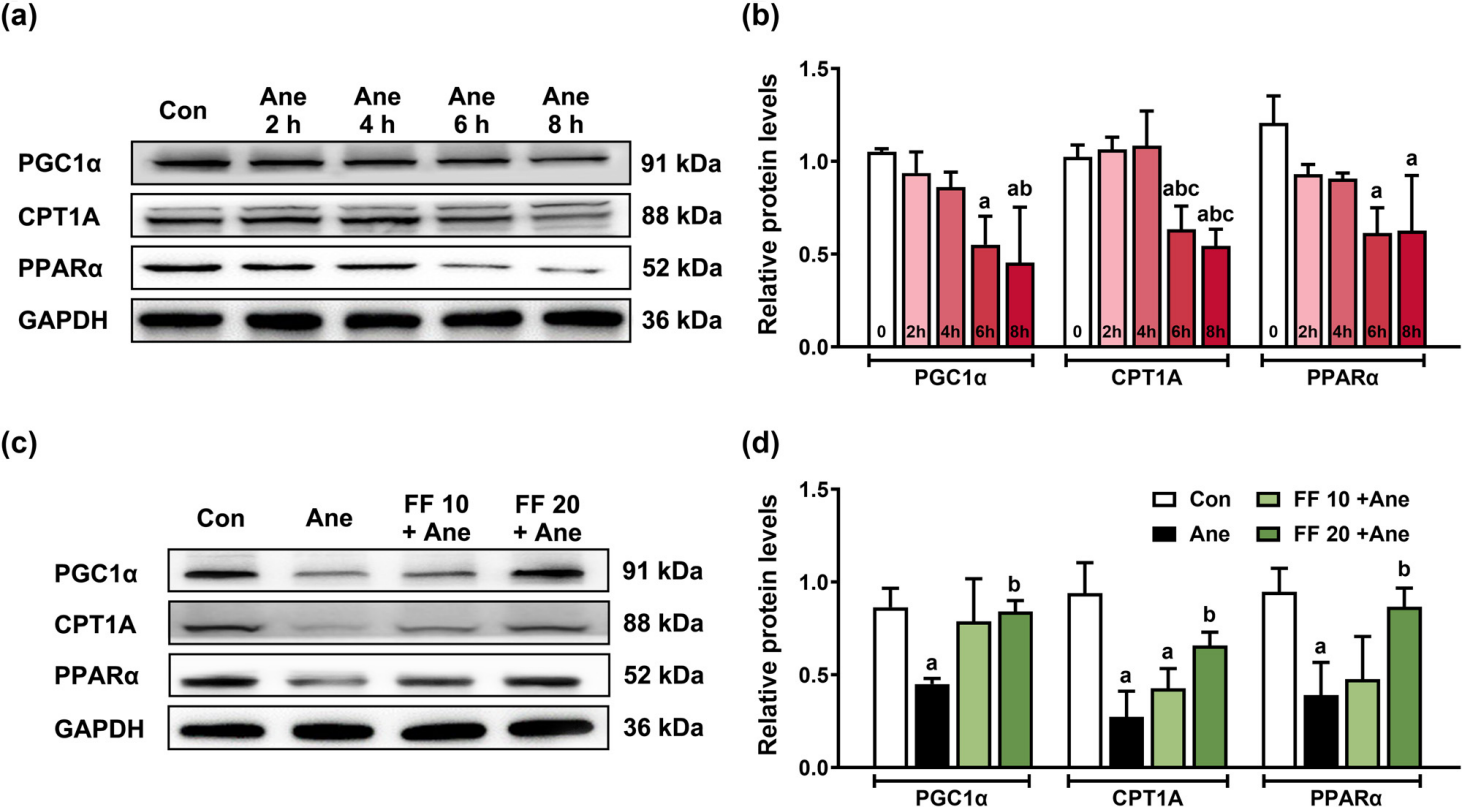
PPARα agonist FF pretreatment rescued the lower levels of fatty acid oxidation-related proteins after long-term isoflurane anesthesia in N2a cells. Isoflurane exposure for 6 h decreases the level of fatty acid oxidation-related proteins, which was reversed by 20 μM FF pretreatment in N2a cells. (a) Relative protein expressions of PGC1α, CPT1A, and PPARα after 2, 4, 6, and 8 h isoflurane anesthesia. (b) The protein quantitation histogram of (a). ^a^ *P* < 0.05, compared with the Con group; ^b^ *P* < 0.05, compared with the Ane 2 h group; ^c^ *P* < 0.05, compared with the Ane 4 h group. (c) Relative protein expression of PGC1α, CPT1A, and PPARα in N2a cells after FF pretreatment and isoflurane anesthesia. (d) The protein quantitation histogram of (c). ^a^ *P* < 0.05, compared with the Con group; ^b^ *P* < 0.05, compared with the Ane group. All data are presented as mean ± SD (*n* = 3/group).

Compared with the Con group, PGC1α, CPT1A, and PPARα protein expression levels were significantly decreased after 1.3% isoflurane intervention for 6 and 8 h rather than 2 and 4 h isoflurane exposure (Figure 2a and b: PGC1α: ^a^ *P* (Ane 6 h) = 0.029, ^a^ *P* (Ane 8 h) = 0.003, ^b^ *P* (Ane 8 h) = 0.043; CPT1A: ^a^ *P* (Ane 6 h) = 0.021, ^a^ *P* (Ane 8 h) = 0.005, ^b^ *P* (Ane 6 h) = 0.011, ^b^ *P* (Ane 8 h) = 0.003, ^c^ *P* (Ane 6 h) = 0.008, ^c^ *P* (Ane 8 h)= 0.002; PPARα: ^a^ *P* (Ane 6 h) = 0.012, ^a^ *P* (Ane 8 h) = 0.014). After that, 6 h isoflurane exposure was selected to establish a prolonged anesthesia cell model for the following *in vitro* experiments.

To verify the effect of PPARα agonist FF pretreatment on fatty acid oxidation-related proteins in the prolonged anesthesia cell model, N2a cells were pretreated with 10 and 20 μM FF 30 min before anesthesia. Compared with the Con group, the protein expression levels of PGC1α (^a^ *P =* 0.028), CPT1A (^a^ *P =* 0.001), and PPARα (^a^ *P =* 0.020) in the Ane group were significantly decreased (Figure 2c and d), and the protein expression levels of CPT1A (^a^ *P =* 0.006) in the FF 10 + Ane group were also significantly decreased (Figure 2c and d). Compared with the Ane group, the protein expressions of PGC1α (^b^ *P =* 0.039), CPT1A (^b^ *P =* 0.033), and PPARα (^b^ *P =* 0.047) in the FF 20 + Ane group were significantly increased (Figure 2c and d).

### 3.3 Effects of PPARα agonist FF on the fatty acid oxidation-related proteins in mice after long-term isoflurane anesthesia

**Figure 3:**
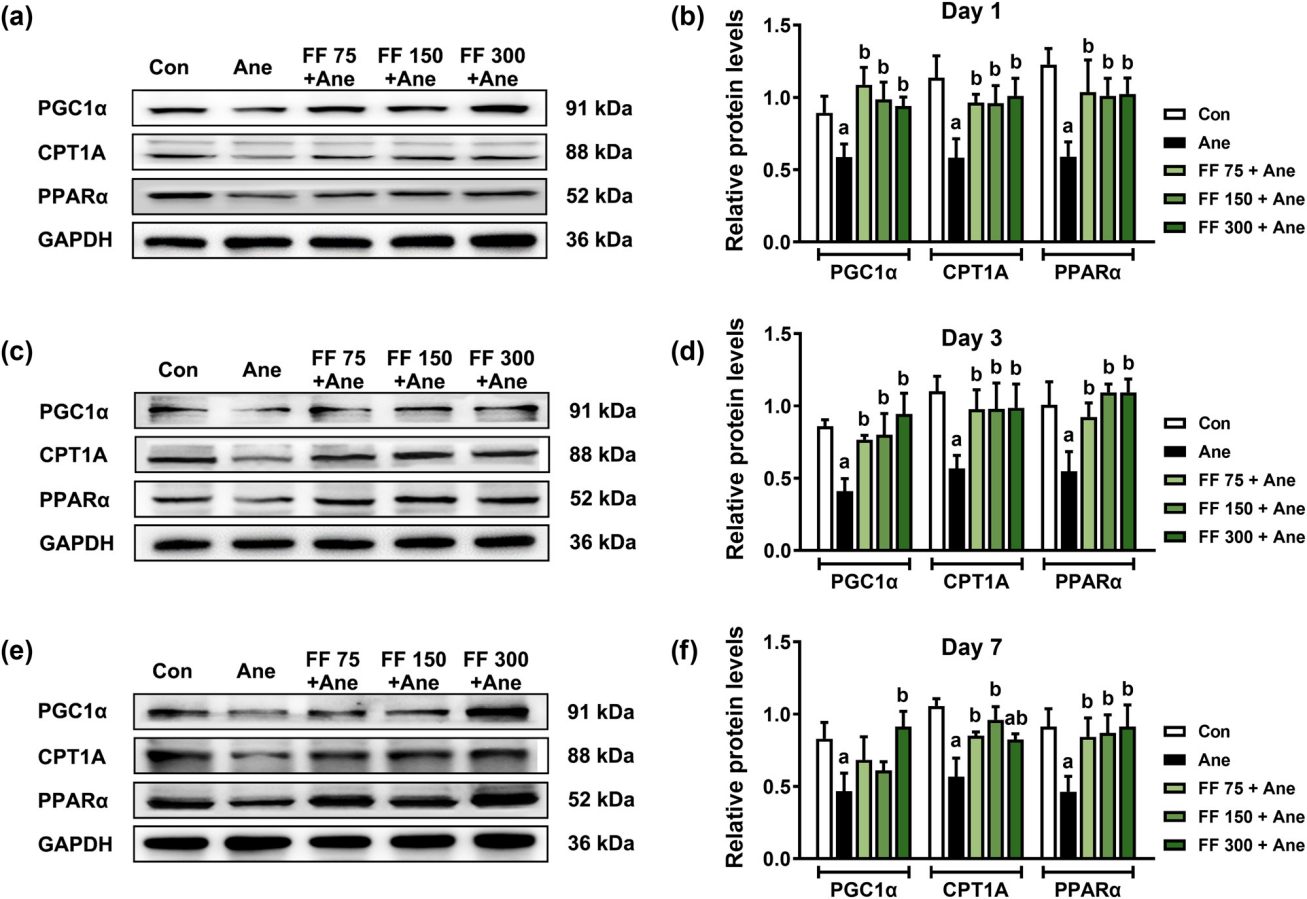
PPARα agonist FF pretreatment ameliorates the decreased level of fatty acid oxidation-related proteins after long-term isoflurane anesthesia in mice. Relative protein expressions of PGC1α, CPT1A, and PPARα on Day 1 (a), 3 (c), and 7 (d) after isoflurane anesthesia. (b) The protein quantitation histogram of (a). (d) The protein quantitation histogram of (c). (f) The protein quantitation histogram of (e). All data are presented as mean ± SD (*n* = 3/group). ^a^ *P* < 0.05, compared with the Con group; ^b^ *P* < 0.05, compared with the Ane group.

Based on the results of *in vitro* experiments, we next explore whether FF upregulates the fatty acid oxidation pathway *in vivo* after long-term isoflurane anesthesia. The mice were given 75, 150, and 300 mg/kg FF 30 min before 6 h isoflurane anesthesia, and the levels of fatty acid oxidation-related proteins PGC1α, CPT1A, and PPARα in the hippocampus of mice on Days 1, 3, and 7 were measured using WB (Figure 3). Compared with the Ane group, the protein levels of PGC1α, CPT1A, and PPARα in the hippocampus of the FF 75 + Ane group, the FF 150 + Ane group, and the FF 300 + Ane group were significantly upregulated on Day 1 (PGC1α: ^b^ *P* (FF 75 + Ane) = 0.002, ^b^ *P* (FF 150 + Ane) = 0.008, ^b^ *P* (FF 300 + Ane) = 0.019; CPT1A: ^b^ *P* (FF 75 + Ane) = 0.031, ^b^ *P* (FF 150 + Ane) = 0.033, ^b^ *P* (FF 300 + Ane) = 0.015; PPARα: ^b^ *P* (FF 75 + Ane) = 0.030, ^b^ *P* (FF 150 + Ane) = 0.044, ^b^ *P* (FF 300 + Ane) = 0.036) and Day 3 (PGC1α: ^b^ *P* (FF 75 + Ane) = 0.017, ^b^ *P* (FF 150 + Ane) = 0.009, ^b^ *P* (FF 300 + Ane) < 0.001; CPT1A: ^b^ *P* (FF 75 + Ane) = 0.046, ^b^ *P* (FF 150 + Ane) = 0.044, ^b^ *P* (FF 300 + Ane) = 0.040; PPARα: ^b^ *P* (FF 75 + Ane) = 0.023, ^b^ *P* (FF 150 + Ane) = 0.002, ^b^ *P* (FF 300 + Ane) = 0.002) after anesthesia (Figure 3a–d). On Day 7 after anesthesia, CPT1A and PPARα protein levels also increased in the FF 75 + Ane group (CPT1A: ^b^ *P* = 0.011; PPARα: ^b^ *P* = 0.046), the FF 150 + Ane group (CPT1A: ^b^ *P* < 0.001; PPARα: ^b^ *P* = 0.030), and the FF 300 + Ane group (CPT1A: ^b^ *P* = 022; PPARα: ^b^ *P* = 0.016), while PGC1α protein levels only in the FF 300+Ane group were significantly higher than that in the Ane group (^b^ *P* = 0.009) (Figure 3e and f).

### 3.4 Effects of PPARα agonist FF on contextual memory of mice after long-term isoflurane anesthesia

**Figure 4:**
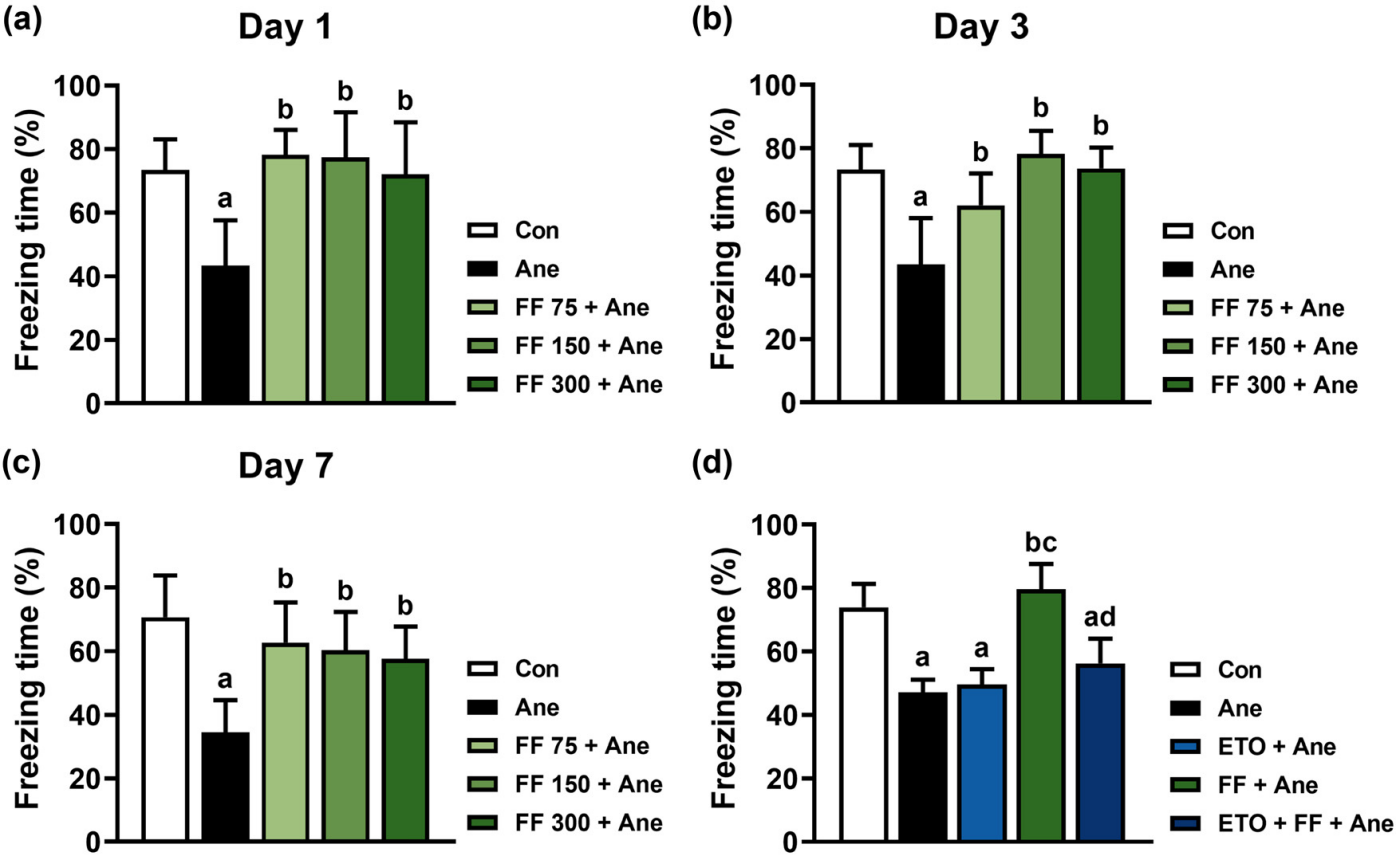
PPARα agonist FF pretreatment improved cognitive function after long-term isoflurane anesthesia in mice. FF pretreatment improves the contextual memory impairments in mice on Day 1 (a), Day 3 (b), and Day 7 (c) after long-term isoflurane anesthesia. All data are presented as mean ± SD (*n* = 6/group). ^a^ *P* < 0.05, compared with the Con group; ^b^ *P* < 0.05, compared with the Ane group; ^c^ *P* < 0.05, compared with the FF 75 + Ane group. (d) CPT1A inhibitor ETO blocks the neuroprotective effect of FF on anesthesia-induced memory impairment in mice. All data are presented as mean ± SD (*n* = 6/group). ^a^ *P* < 0.05, compared with the Con group; ^b^ *P* < 0.05, compared with the Ane group; ^c^ *P* < 0.05, compared with the ETO + Ane group; ^d^ *P* < 0.05, compared with the FF + Ane group.

In order to determine the effects of FF pretreatment on cognitive impairment after long-term isoflurane anesthesia, the contextual memory of mice was detected using the fear conditioning system. As shown in Figure 4a–c, in the contextual fear conditioning system, the percentage of freezing time of mice in the FF 75 + Ane group, the FF 150 + Ane group, and the FF 300 + Ane group increased significantly than that of the Ane group on Day 1 (^b^ *P* (FF 75 + Ane) < 0.001, ^b^ *P* (FF 150 + Ane) = 0.001, ^b^ *P* (FF 300 + Ane) = 0.007), Day 3 (^b^ *P* (FF 75 + Ane) = 0.028, ^b^ *P* (FF 150 + Ane) < 0.001, ^b^ *P* (FF 300 + Ane) < 0.001), and Day 7 (^b^ *P* (FF 75 + Ane) = 0.003, ^b^ *P* (FF 150 + Ane) = 0.008, ^b^ *P* (FF 300 + Ane) = 0.021) after anesthesia.

To further validate whether the fatty acid oxidation pathway is involved in the neuroprotective effect of FF on anesthesia-induced memory impairment, 25 mg/kg CPT1A inhibitor ETO was intraperitoneally injected into mice for 3 days before anesthesia. Based on the results in Figure 4a–c, we set the dose of FF to 300 mg/kg to reach the maximum neuroprotective effect. As shown in Figure 4d, in the contextual fear conditioning test, there were no differences among the Ane group, the ETO + Ane group, and the ETO + FF + Ane group, whereas the percentage of freezing time in the FF + Ane group increased significantly compared with the Ane group (^b^ *P <* 0.001). Remarkably, the percentage of freezing time of the ETO + FF + Ane group was significantly lower than that in the FF + Ane group (^d^ *P* < 0.001), which suggests that after blocking fatty acid oxidation by ETO, FF failed to improve the memory impairment after long-term isoflurane anesthesia.

## 4 Discussion

**Figure 5:**
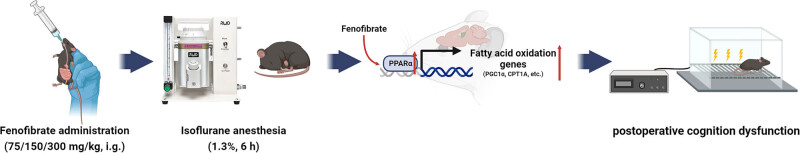
Pretreatment of PPARα agonist FF can protect against 6 h isoflurane anesthesia-induced POCD by enhancing fatty acid oxidation.

This study focused on the memory impairment induced by 6 h long-term isoflurane anesthesia, simulating the process of “POCD,” as shown in Figure 5. We found that long-term isoflurane anesthesia resulted in contextual memory impairment in mice lasting 7 days after anesthesia. Meanwhile, fatty acid oxidation-related protein (PGC1α, CPT1A, PPARα) expression levels decreased in the hippocampus of POCD mice. We administered PPARα agonist FF to POCD models in mice and N2a cells before anesthesia to explore its neuroprotective effects. Both *in vitro* and *in vivo* experiments showed that FF pretreatment reversed the decrease of related protein expression levels. Furthermore, our results showed that a supplement of FF could improve impairments of contextual memory after long-term isoflurane anesthesia in mice. To thoroughly explore whether FF protects against cognitive impairment after anesthesia by enhancing the fatty acid oxidation pathway, a CPT1A inhibitor, ETO, was used to block the pathway. Results showed that ETO pretreatment failed to despair the memory of POCD mice but could block the protective effect of FF. These results further suggested that FF protects memory impairment after isoflurane anesthesia by enhancing the fatty acid oxidation pathway.

Some studies concerning the mechanisms of POCD have focused on disorders of fatty acid oxidation. Qian and Wang investigated the serum metabolism of POCD and the results revealed that fatty acid metabolism and lipid metabolism were considerably altered [17]. Research in rodents showed that inhibition of fatty acid oxidation enhanced oxidative stress and induced Aβ deposition *in vivo* and *in vitro* [18]. Meanwhile, the protein levels of PPARα and PGC1α in the hippocampus decreased significantly after anesthesia, which was consistent with the findings of Yong et al. that inhalation of 1.3% isoflurane for 3 h for 3 days in newborn rats decreased the expression level of PPARα in the hippocampus [19]. In POCD mice, the expression level of PGC1α in the hippocampus also decreased significantly [20] when induced by internal fixation of tibia fracture along with continuous inhalation of 1.5% isoflurane for 4 h. PPARα is demonstrated to be critical in fatty acid oxidation, and PGC1α is a member of the transcriptional coactivator family and can interact with PPARα to regulate mitochondrial fatty acid oxidation-related genes [21], such as CPT1A and CPT2 [22,23]. In this study, CPT1A, the key mitochondrial fatty acid oxidation enzyme, was also significantly decreased after long-term isoflurane anesthesia.

PPARα is altered in multiple kinds of neurodegenerative/neurodevelopmental and psychiatric disorders, suggesting that this receptor could be a viable target in novel therapeutic strategies. FF, one of the PPARα agonists, is a derivative of fibric acid and is commonly applied in the treatment of adults with dyslipidemia, primary hypertriglyceridemia, and hypercholesterolemia [24]. Previous studies have reported that FF could decrease inflammation and oxidative stress accumulation to mediate neuroprotective benefits in mice models of AD, PD, and brain damage [13,25,26]. The present study showed that FF could reverse the downregulation of fatty acid oxidation-related proteins both *in vivo* and *in vitro* in POCD models. In line with our results, Goto et al. showed that PPARα agonist FF upregulates PGC1α transcription [27]. Khorolskaya et al. also found that oral administration of 0.3% FF induced mitochondrial fatty acid β oxidation in mice and increased protein expression of PPARα, PGC1α, and CPT1A [28]. The promoter region of CPT1A has a peroxisome proliferator response element (PPRE), which when combined with PPARα can upregulate the expression of CPT1A [23]. However, PPARα and PGC1α regulate CPT1A independently in rodents, with PPARα binding to PPRE in the second intron of CPT1A and PGC1α binding to PPRE in the first intron of CPT1A [29]. Therefore, the reversal effect of FF on the CPT1A decrease after long-term isoflurane anesthesia may be attributed to the direct regulation of CPT1A expression by activating PPARα, or to the indirect upregulation of CPT1A via PGC1α.

PPARα is reported to be critical in cognition and emotions in the study of the last decade [30]. Roy et al. showed that the introduction of PPARα to the hippocampus of PPARα-KO mice improved spatial learning and memory [9]. Currently, multiple PPARα natural and synthetic agonists are being tested in clinical studies or experimental models of neurodegenerative/psychiatric diseases [31]. PPARα endogenous agonist PEA is an anti-inflammatory compound clinically tested for its neuroprotective effects in AD and PD [32]. In the research of Luo et al., PPARα agonists Gemfibrozil and Wy14643 could ameliorate the deposition of Aβ proteins and improve cognitive dysfunction in AD mice [33]. Similarly, the PPARα agonist FF, as a selective synthetic agonist of PPARα, has been demonstrated to exert significant ameliorating effects on cognitive impairment in animal experiments and clinical patients of AD and PD [30,34]. Consistent with our research, pretreatment with FF has preventive effects on neurovascular and cognitive consequences in many other nervous system diseases, such as brain ischemia and irradiation, and type 2 diabetes [12,35,36]. However, the fundamental mechanisms of FF’s neuroprotective effects remain ambiguous. These neuroprotective effects might be attributable to FF’s antioxidant and anti-inflammatory properties as well as to advantageous effects on glucose homeostasis and lipid metabolism [13,37,38]. The PPARα agonist FF might be a novel target for therapeutic schemes of cognitive disorders. Our research demonstrated that FF protects memory impairment after isoflurane anesthesia by enhancing the fatty acid oxidation pathway in mice. In terms of the mode of administration, in our study, we administered FF to mice through intragastric infusion at a single dose before anesthesia; whereas, other researchers chose to administer FF for several days continuously, such as 200 mg/kg/day for 14 days in cardiopulmonary bypass surgery mice and twice a day during 72 h or 7 days in stroke mice [12,39]. The time of administration also varied in many studies, among which Ouk et al. administered poststroke orally and Barbiero et al. gave FF to mice 1 h after PD model establishment [12,13]. On the basis of existing studies and our results, taking an oral drug prophylactically at a low and single dose might accelerate the translation of basic science to clinical practice.

There are some limitations to our research: (1) we investigated the neuroprotective effect of FF on cognitive impairments through the fatty acid oxidation pathway in the POCD model at the protein level. Considering PPARα is an important transcription factor, more research should be carried out at the transcriptional level to further verify the regulatory effects of FF on fatty acid oxidation-related proteins. (2) In each group, the sample sizes for protein detection were less than those for behavior assessment. (3) As shown in Figure 4d, we evaluated cognitive functions only on Day 3 after anesthesia to assess the effect of ETO on FF’s neuroprotection; however, mice’s learning and memory on Days 1 and 7 should also be detected. (4) Fatty acid oxidation is closely related to oxidative stress. We should further explore the composition and quantity of fatty acids as well as markers of oxidative stress such as SOD, H_2_O_2_, and MDA with FF pretreatment following anesthesia. In this way, our study will be more convincing. Further study should be performed to solve the inadequacies listed above.

## 5 Conclusion

The present study demonstrated the protective effect of PPARα agonist FF on memory impairment after long-term isoflurane anesthesia and identified the role of fatty acid oxidation-related proteins in the process of POCD. This finding could help to improve our understanding of the amnestic effect of isoflurane and to provide new strategies for it.

## References

[1] Lee YM, Song BC, Yeum KJ. Impact of volatile anesthetics on oxidative stress and inflammation. Biomed Res Int. 2015;2015:242709. 10.1155/2015/242709 PMC4458520 26101769

[2] Eckel RH, Grundy SM, Zimmet PZ. The metabolic syndrome. Lancet. 2005;365(9468):1415–28. 10.1016/S0140-6736(05)66378-7 15836891

[3] Fang X, Xia T, Xu F, Wu H, Ma Z, Zhao X, et al. Isoflurane aggravates peripheral and central insulin resistance in high-fat diet/streptozocin-induced type 2 diabetic mice. Brain Res. 2020;1727:146511. 10.1016/j.brainres.2019.146511 31672472

[4] Peng L, Fang X, Xu F, Liu S, Qian Y, Gong X, et al. Amelioration of hippocampal insulin resistance reduces tau hyperphosphorylation and cognitive decline induced by isoflurane in mice. Front Aging Neurosci. 2021;13:686506. 10.3389/fnagi.2021.686506 PMC8425557 34512303

[5] Kahn CR, Wang G, Lee KY. Altered adipose tissue and adipocyte function in the pathogenesis of metabolic syndrome. J Clin Invest. 2019;129(10):3990–4000. 10.1172/JCI129187 PMC6763230 31573548

[6] Liu F, Rainosek SW, Frisch-Daiello JL, Patterson TA, Paule MG, Slikker W, et al. Potential adverse effects of prolonged sevoflurane exposure on developing monkey brain: from abnormal lipid metabolism to neuronal damage. Toxicol Sci. 2015;147(2):562–72. 10.1093/toxsci/kfv150 PMC4707202 26206149

[7] Feinkohl I, Winterer G, Pischon T. Associations of dyslipidaemia and lipid-lowering treatment with risk of postoperative cognitive dysfunction: a systematic review and meta-analysis. J Epidemiol Community Health. 2018;72(6):499–506. 10.1136/jech-2017-210338 29437865

[8] Ebert D, Haller RG, Walton ME. Energy contribution of octanoate to intact rat brain metabolism measured by 13C nuclear magnetic resonance spectroscopy. J Neurosci. 2003;23(13):5928–35. 10.1523/JNEUROSCI.23-13-05928.2003 PMC6741266 12843297

[9] Roy A, Jana M, Corbett GT, Ramaswamy S, Kordower JH, Gonzalez FJ, et al. Regulation of cyclic AMP response element binding and hippocampal plasticity-related genes by peroxisome proliferator-activated receptor α. Cell Rep. 2013;4(4):724–37. 10.1016/j.celrep.2013.07.028 PMC3804033 23972989

[10] Moreno S, Farioli-Vecchioli S, Cerù MP. Immunolocalization of peroxisome proliferator-activated receptors and retinoid X receptors in the adult rat CNS. Neuroscience. 2004;123(1):131–45. 10.1016/j.neuroscience.2003.08.064 14667448

[11] Schoonjans K, Martin G, Staels B, Auwerx J. Peroxisome proliferator-activated receptors, orphans with ligands and functions. Curr Opin Lipidol. 1997;8(3):159–66. 10.1097/00041433-199706000-00006 9211064

[12] Ouk T, Gautier S, Pétrault M, Montaigne D, Maréchal X, Masse I, et al. Effects of the PPAR-α agonist fenofibrate on acute and short-term consequences of brain ischemia. J Cereb Blood Flow Metab. 2014;34(3):542–51. 10.1038/jcbfm.2013.233 PMC3948136 24398933

[13] Barbiero JK, Santiago R, Tonin FS, Boschen S, da Silva LM, Werner MF, et al. PPAR-α agonist fenofibrate protects against the damaging effects of MPTP in a rat model of Parkinson's disease. Prog Neuropsychopharmacol Biol Psychiatry. 2014;53:35–44. 10.1016/j.pnpbp.2014.02.009 24593945

[14] Jiang S, Uddin MJ, Yu X, Piao L, Dorotea D, Oh GT, et al. Peroxisomal Fitness: A Potential Protective Mechanism of Fenofibrate against High Fat Diet-Induced Non-Alcoholic Fatty Liver Disease in Mice. Diabetes Metab J. 2022;46(6):829–42. 10.4093/dmj.2021.0274 PMC9723204 35746892

[15] Sohn M, Kim K, Uddin MJ, Lee G, Hwang I, Kang H, et al. Delayed treatment with fenofibrate protects against high-fat diet-induced kidney injury in mice: the possible role of AMPK autophagy. Am J Physiol Renal Physiol. 2017;312(2):F323–34. 10.1152/ajprenal.00596.2015 27465995

[16] Javanbakht S, Shaabani A. Carboxymethyl cellulose-based oral delivery systems. Int J Biol Macromol. 2019;133:21–9. 10.1016/j.ijbiomac.2019.04.079 30986470

[17] Qian G, Wang Y. Serum metabolomics of early postoperative cognitive dysfunction in elderly patients using liquid chromatography and Q-TOF mass spectrometry. Oxid Med Cell Longev. 2020;2020:8957541. 10.1155/2020/8957541 PMC7007934 32082482

[18] Shi Y, Sun X, Sun Y, Hou L, Yao M, Lian K, et al. Elevation of cortical C26:0 due to the decline of peroxisomal β-oxidation potentiates amyloid β generation and spatial memory deficits via oxidative stress in diabetic rats. Neuroscience. 2016;315:125–35. 10.1016/j.neuroscience.2015.11.067 26687434

[19] Yong J, Yan L, Wang J, Xiao H, Zeng Q. Effects of compound 21, a non‑peptide angiotensin II type 2 receptor agonist, on general anesthesia‑induced cerebral injury in neonatal rats. Mol Med Rep. 2018;18(6):5337–44. 10.3892/mmr.2018.9602 PMC6236271 30365086

[20] Zhao Z, Yao M, Wei L, Ge S. Obesity caused by a high-fat diet regulates the Sirt1/PGC-1α/FNDC5/BDNF pathway to exacerbate isoflurane-induced postoperative cognitive dysfunction in older mice. Nutr Neurosci. 2020;23(12):971–82. 10.1080/1028415X.2019.1581460 30794116

[21] Scarpulla RC, Vega RB, Kelly DP. Transcriptional integration of mitochondrial biogenesis. Trends Endocrinol Metab. 2012;23(9):459–66. 10.1016/j.tem.2012.06.006 PMC3580164 22817841

[22] Zhang Y, Ma K, Song S, Elam MB, Cook GA, Park EA. Peroxisomal proliferator-activated receptor-gamma coactivator-1 alpha (PGC-1 alpha) enhances the thyroid hormone induction of carnitine palmitoyltransferase I (CPT-I alpha). J Biol Chem. 2004;279(52):53963–71. 10.1074/jbc.M406028200 15469941

[23] Lamichane S, Dahal Lamichane B, Kwon SM. Pivotal Roles of Peroxisome Proliferator-Activated Receptors (PPARs) and their signal cascade for cellular and whole-body energy homeostasis. Int J Mol Sci. 2018;19(4):949. 10.3390/ijms19040949 PMC5979443 29565812

[24] Keating GM, Croom KF. Fenofibrate: a review of its use in primary dyslipidaemia, the metabolic syndrome and type 2 diabetes mellitus. Drugs. 2007;67(1):121–53. 10.2165/00003495-200767010-00013 17209672

[25] Deplanque D, Gelé P, Pétrault O, Six I, Furman C, Bouly M, et al. Peroxisome proliferator-activated receptor-alpha activation as a mechanism of preventive neuroprotection induced by chronic fenofibrate treatment. J Neurosci. 2003;23(15):6264–71. 10.1523/JNEUROSCI.23-15-06264.2003 PMC6740545 12867511

[26] Inestrosa NC, Carvajal FJ, Zolezzi JM, Tapia-Rojas C, Serrano F, Karmelic D, et al. Peroxisome proliferators reduce spatial memory impairment, synaptic failure, and neurodegeneration in brains of a double transgenic mice model of Alzheimer's disease. J Alzheimers Dis. 2013;33(4):941–59. 10.3233/JAD-2012-120397 23109558

[27] Goto T, Hirata M, Aoki Y, Iwase M, Takahashi H, Kim M, et al. The hepatokine FGF21 is crucial for peroxisome proliferator-activated receptor-α agonist-induced amelioration of metabolic disorders in obese mice. J Biol Chem. 2017;292(22):9175–90. 10.1074/jbc.M116.767590 PMC5454100 28404815

[28] Khorolskaya VG, Gureev AP, Shaforostova EA, Laver DA, Popov VN. The fenofibrate effect on genotoxicity in brain and liver and on the expression of genes regulating fatty acids metabolism of mice. Biomed Khim. 2019;65(5):388–97. 10.18097/PBMC20196505388 31666411

[29] Song S, Attia RR, Connaughton S, Niesen MI, Ness GC, Elam MB. et al. Peroxisome proliferator activated receptor alpha (PPARalpha) and PPAR gamma coactivator (PGC-1alpha) induce carnitine palmitoyltransferase IA (CPT-1A) via independent gene elements. Mol Cell Endocrinol. 2010;325(1–2):54–63. 10.1016/j.mce.2010.05.019 PMC3160239 20638986

[30] Nisbett KE, Pinna G. Emerging therapeutic role of PPAR-α in cognition and emotions. Front Pharmacol. 2018;9:998. 10.3389/fphar.2018.00998 PMC6190882 30356872

[31] Wójtowicz S, Strosznajder AK, Jeżyna M, Strosznajder JB. The novel role of PPAR alpha in the brain: promising target in therapy of alzheimer's disease and other neurodegenerative disorders. Neurochem Res. 2020;45(5):972–88. 10.1007/s11064-020-02993-5 PMC7162839 32170673

[32] Tufano M, Pinna G. Is there a future for PPARs in the Treatment of neuropsychiatric disorders? Molecules. 2020;25(5):1062. 10.3390/molecules25051062 PMC7179196 32120979

[33] Luo R, Su LY, Li G, Yang J, Liu Q, Yang LX, et al. Activation of PPARA-mediated autophagy reduces Alzheimer disease-like pathology and cognitive decline in a murine model. Autophagy. 2020;16(1):52–69. 10.1080/15548627.2019.1596488 PMC6984507 30898012

[34] Di Giacomo E, Benedetti E, Cristiano L, Antonosante A, d'Angelo M, Fidoamore A, et al. Roles of PPAR transcription factors in the energetic metabolic switch occurring during adult neurogenesis. Cell Cycle. 2017;16(1):59–72. 10.1080/15384101.2016.1252881 PMC5270516 27860527

[35] Ramanan S, Kooshki M, Zhao W, Hsu FC, Riddle DR, Robbins ME. The PPARalpha agonist fenofibrate preserves hippocampal neurogenesis and inhibits microglial activation after whole-brain irradiation. Int J Radiat Oncol Biol Phys. 2009;75(3):870–7. 10.1016/j.ijrobp.2009.06.059 PMC2789462 19801103

[36] Rizk FH, Soliman NA, Heabah NA, Abdel Ghafar MT, El-Attar SH, Elsaadany A. Fenofibrate improves cognitive impairment induced by high-fat high-fructose diet: A possible role of irisin and heat shock proteins. ACS Chem Neurosci. 2022;13(12):1782–9. 10.1021/acschemneuro.2c00186 35652596

[37] Assaf N, El-Shamarka ME, Salem NA, Khadrawy YA, El Sayed NS. Neuroprotective effect of PPAR alpha and gamma agonists in a mouse model of amyloidogenesis through modulation of the Wnt/beta catenin pathway via targeting alpha- and beta-secretases. Prog Neuropsychopharmacol Biol Psychiatry. 2020;97:109793. 10.1016/j.pnpbp.2019.109793 31669201

[38] Ramanan S, Kooshki M, Zhao W, Hsu FC, Robbins ME. PPARalpha ligands inhibit radiation-induced microglial inflammatory responses by negatively regulating NF-kappaB and AP-1 pathways. Free Radic Biol Med. 2008;45(12):1695–704. 10.1016/j.freeradbiomed.2008.09.002 PMC2648135 18852043

[39] Ouk T, Amr G, Azzaoui R, Delassus L, Fossaert E, Tailleux A, et al. Lipid-lowering drugs prevent neurovascular and cognitive consequences of cardiopulmonary bypass. Vascul Pharmacol. 2016;80:59–66. 10.1016/j.vph.2015.12.005 26779598

